# Extracorporeal Cardiopulmonary Resuscitation Guided by End-Tidal Carbon Dioxide—a Porcine Model

**DOI:** 10.1007/s12265-022-10210-7

**Published:** 2022-03-14

**Authors:** Carl-Henrik Ölander, Per Vikholm, Rickard Lindblom, Petter Schiller, Laila Hellgren

**Affiliations:** Department of Cardiothoracic Surgery, Uppsala University Hospital, SE-75185 Uppsala, Sweden

**Keywords:** Cardiopulmonary resuscitation, End-tidal carbon dioxide, Extracorporeal cardiopulmonary resuscitation, Microdialysis, Prognostic factors

## Abstract

Extracorporeal membrane cardiopulmonary resuscitation (ECPR) during cardiopulmonary resuscitation (CPR) for selected cases and end-tidal carbon dioxide (ETCO_2_) could be used to guide initiation of ECPR. Ventricular fibrillation was induced in 12 pigs and CPR was performed until ETCO_2_ fell below 10 mmHg; then, ECPR was performed. Animals were divided into group short (G_Short_) and group long (G_Long_), according to time of CPR. Carotid blood flow was higher (*p* = 0.02) and mean arterial blood pressure lower in G_Long_ during CPR (*p* < 0.05). B-Lactate was lower and pH higher in G_Short_ (*p* < 0.01). In microdialysis lactate-pyruvate ratio, glycerol and glutamate increased in both groups during CPR, but considerably in G_Long_ (*p* < 0.01). No difference could be seen in histopathology of the brain or kidney post-ECPR. No apparent histological differences of tissue damage in brains or levels of S100B in plasma were detected between groups. This might suggest that ETCO_2_ could be used as a marker for brain injury following ECPR.

**Graphical abstract:**

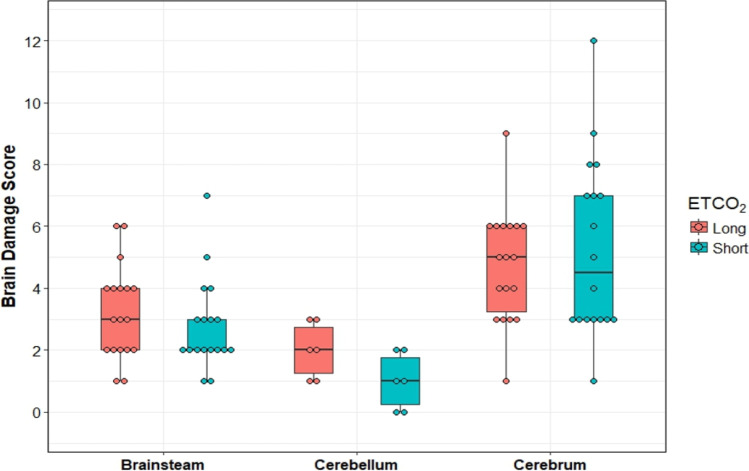

## Introduction

Cardiac arrest still carries a high mortality rate with survival to discharge only 10–15% in witnessed arrests [1, 2] and close to 0% if unwitnessed [3]. The introduction of extracorporeal cardiopulmonary resuscitation (ECPR) has increased survival in witnessed arrests [4–6], but neurologic and renal complications remain high [7].

ECPR treatment is rapidly increasing in numbers. However, the treatment is expensive, resource-demanding, and associated with severe complications and therefore its usage must be optimised. Existing selection criteria which predict a favourable patient outcome, based on retrospective studies, have identified shockable rhythm, shorter low-flow time, higher pH, and lower B-lactate [8–10]. In spite of some progress made in identification of favourable predictors, mortality and morbidity after ECPR are still unacceptably high [11]. Factors that can facilitate the clinical decision-making in ECPR are needed.

ETCO_2_ correlates with cardiac output and cerebral blood flow during CPR and is a part of the CPR algorithm for guidance of compressions, detection, and chances of a return of spontaneous circulation (ROSC) [12–14]. Establishing the quality of CPR and the patient’s current physiologic condition is of high importance before initiation of ECPR, in order to estimate chances of a long-term outcome when circulation is restored on extracorporeal circuit. ETCO_2_ is easily obtained since it is already part of the conventional CPR algorithm.

We hypothesised that the level of ETCO_2_—as a complement to time—can be used to guide initiation of ECPR treatment in an experimental porcine model of cardiac arrest. This was tested in an experimental porcine model of cardiac arrest, where brain and kidney injury were studied after CPR and ECPR.

## Material and Methods

### Animal Model

This study was approved by Uppsala Ethical Committee on Laboratory Animal Research. The experiment was conducted in accordance with the European Community Standards on Care and Use of Laboratory animals. The protocol, hypothesis, and design were prepared before the study. All data and the study protocol are stored in the laboratory facilities.

### Animal Preparation

The study included 12 male pigs of Swedish country breed, with a mean weight of 51 (range 46–60) kg. Pigs were considered healthy upon arrival to the laboratory.

Pigs were anesthetised by subcutaneous injection of xylazine (Rompun®, Bayer A/S, Denmark) and tiletamin/zolzepam (Zoletil 100®, Virbac S.A., France). Maintenance of anaesthesia was achieved by using intravenous (IV) infusion of buffered glucose carrier (Rehydrex® 25 mg/ml, Fresenius Kabi AB, Sweden), ketamine 30 mg/kg/h (Ketaminol vet®, Intervet AB, Sweden), fentanyl 0.04 mg/kg/h (Fentanyl, B Braun Medical AB, Sweden), and midazolam 0.1 mg/kg/h (Midazolam Hameln, AlgolPharma AB, Sweden). Muscular blockade was obtained using rocuronium 2.5 mg/kg/h (Esmeron, MSD AB, Sweden).

Surgical tracheostomy was performed, and the animals were normo-ventilated (mainstream capnography). A volume-controlled mode with peak end-expiratory pressure setting of 5 cmH_2_O and FiO_2_ of 0.3, using Siemens Servo-I ventilator (Maquet GmbH & Co. Germany) was used.

The right external jugular vein was catheterised with a pulmonary artery catheter and a central venous line (Criticath™ Pulmonary Artery/Thermodilution Catheter, Singapore and BD Careflow 17G BD). In a retrograde direction, a third (3 Fr) catheter was placed in the right external carotid artery. A femoral line was placed and used for blood sampling, drug administration, and circulatory monitoring. Blood flow in the left internal carotid artery was registered by an ultrasonic probe (P201, 3RB246, Transoncic Systems Inc, USA). The urinary bladder was catheterised using a silicone catheter.

Two drill hole craniotomies were performed. A 3 Fr catheter was placed in the sagittal vein for blood sampling and pressure measurements. Intracranial pressure was measured by a modified cholangial catheter placed in the right lateral cerebral ventricle. In a third drill hole in the right frontal hemisphere, a hollow bolt (GSM Licox, Germany) was placed, through which a microdialysis catheter (70 Microdialysis Bolt Catheter; M Dialysis AB, Stockholm, Sweden) with 10 mm polyamide membrane and a 20 kDa cutoff (M Dialysis AB) was inserted into the cerebral parenchyma. The catheter was perfused with microdialysis fluid (Perfusion Fluid CNS, containing Na^+^ 148 mM, Ca^2+^ 1.2 mM, Mg^2+^0.9 mM, K^+^ 2.7 mM, and Cl^−^ 155 mM; M Dialysis AB) at a flow rate of 1μl/min, using a 106 MD pump (M Dialysis AB).

The right femoral artery and vein were cannulated through surgical cut-down, using 16 and 19 F cannulas (Maquet, Germany), respectively. Cannulas were flushed with saline and clamped. An intravenous bolus of 5000 IU of heparin (Heparin, LEO Pharma AB, Sweden) was administered and repeated every 60 min.

### Experimental Protocol

The protocol included four phases: (1) preparation and baseline measurements, (2) CPR until ETCO_2_ reached 10 mmHg, (3) ECPR for 180 min, and (4) termination of experiment.

The animals were fixated using a vacuum mattress, and the LUCAS™ Chest Compression System (Physio-Control Inc./Jolife AB, Sweden) was placed on the caudal 1/3 of the sternum. Ventricular fibrillation was induced (Slide regulator SD242, Matsunaga MF Co. Ltd, Japan) and mechanical CPR was started and commenced according to ILCOR guidelines. Doses of adrenalin (epinephrine) were set to 0.1 mg/kg (Martindale Pharma, UK). Analysis of arterial blood (B) gases was performed using ABL 500 Radiometer, Medical ApS, Denmark. Samples were drawn every 15 min and 100 ml of buffer (Tribonat, Fresenius Kabi, Sweden) was administered intravenously, if pH<7.2. No alterations were made in ventilation mode. FiO_2_ was set to 1.0. Levels of end-tidal carbon dioxide were continuously recorded. When ETCO_2_ levels fell below 10 mmHg for more than 1 min, mechanical CPR was stopped and ECPR was started. The time of CPR was a random effect of the standardised performed CPR and each animals’ individual response to CPR, as measured by ETCO_2_. The two groups were built by random.

Femoral cannulas were connected to an extracorporal membrane oxygenation system (Quadrox-i oxygenator, Maquet, Medtronic BPX-80 Centrifugal BIO-Pump Plus, Germany and 3T Heater-Cooler Unit, LivaNova PLC, UK), pre-primed with ringer-acetate (Ringer-Acetate, Fresenius Kabi, Sweden). Initial blood flow was the equivalent of baseline cardiac output measurement. ECMO-FiO_2_ was 1.0 and gas flow was set to return arterial carbon dioxide levels to normal. Ventilator-FiO_2_ was returned to 0.3. Normothermia was maintained by a heater-cooler unit and body temperature was measured. If the carotid mean arterial pressure (MAP) fell below 50 mmHg, an intravenous infusion of norepinephrine, max dose of 0.45 mcg/kg/min, was started. At 180 min of ECPR, treatment was terminated, and euthanasia was performed. The arterial cannula of the ECMO system was disconnected, and the animal was bled out through the venous cannula.

Following craniotomy, the brain was harvested for histologic examination. A right-sided nephrectomy was performed for the same purpose.

### Measured Parameters and Analysis

Baseline measurements and cardiac output were recorded (Dräger Infinity Delta) using the thermodilution technique. Arterial blood (B) gases were analysed (ABL 500 Radiometer, Medical ApS, Denmark) every 15 min. Hemodynamic measurements were recorded and blood and urine sampling repeated every 30 min during extracorporeal treatment. Microdialysis vials were exchanged every 15 min.

P-nGAL was analysed using “Pig nGAL ELISA-kit” (Bio Porto Diagnostics A/S, Denmark). The cerebral microdialysate was analysed for glucose, lactate, pyruvate, glutamate, glycerol, and urea (ISCUSflex analyser, M Dialysis AB). The lactate-pyruvate ratio was calculated. Urea was used as an endogenous control for microdialysis catheter performance. Porcine IL6 and TNF-alpha using commercially available microtiter plate–based ELISA kits (DY686 and DY690, R&D Systems, Minneapolis, MN, USA) were used, according to the manufacturer’s instructions. The total coefficient of variations (CVs) of the ELISAs was approximately 6%. Porcine S100B was analysed by CanAg® S100B EIA (Fujirebio, Diagnostics, Gothenburg, Sweden).

Kidneys and brains were processed, and tissues were fixated in formaldehyde and blocked in paraffin to obtain three large sections (kidney cranial pole, middle, and caudal pole including cortex and medulla; and from the brain cortex, cerebellum and brain stem). Sections of 4μm were stained with hematoxylin and eosin.

The histopathological changes for kidneys were grouped into (1) distension of Bowman’s space, (2) hyperaemia/pooling of blood in the lumen of vessels, (3) damage to tubular epithelium to cortex and medulla, and (4) other findings such as focal hemorrhage and fibrin in the vessels. The histopathological changes for the brains were grouped into (1) red neurons (ischemic damage), (2) neutrophil vacuolation, (3) perivascular oedema, and (4) hemorrhages (extravasation of blood into the brain white or grey matter). A brain damage score was calculated in which lesions were described according to pathologist-standardised protocol, and scored 0 points if absent, and 1–3 points due to degree of inter-individual severity and extension.

Histopathological evaluations and the blocking of the organs were performed blindly by a veterinary pathologist, without the knowledge of the CPR time.

### Statistical Analysis

Subjects were divided into group short (G_Short_) (*n*=6) and group long (G_Long_) (*n*=6), according to time of CPR until ETCO_2_ fell to 10mmHg. The programme R (v3.4.0) was used for statistical analysis. Due to the small sample size, the hemodynamic values, laboratory parameters, and histological brain damage score are presented as median with inter-quartile range (IQR).

Using the Mann-Whitney *U*-test, the two groups were compared regarding differences in baseline. In order to minimise the impact of differences in baseline values, and random variability on the repeated measures between the two groups during CPR and ECPR, a repeated-measurement mixed effect model approach was used (R-package lme4 (v. 1.1-15)). The group of animals was considered fixed, and the individual subjects were treated as random effects. For each parameter, two models were created. The first model only used random intercept and the second both random intercept and slope for each subject. Using ANOVA, the models were compared. If the model with both random intercept and slope did not add information, the more conservative model (only random intercept) was chosen. Normal distribution of data was obtained by graphical analysis of the residuals for each parameter.

The two groups were also compared regarding cerebral histological changes in each region using a similar mixed effect model. However, instead of change over time, the difference between the regions was compared for each group with random intercept and slope for each subject. This was done for both the total brain damage score and each sub-score. *p*-values <0.05 were considered statistically significant.

## Results

All animals (*n*=12) were included in strict order upon arrival from a farmer; no animal was excluded. All animals survived the experiment and constituted the study group.

At baseline, no differences were found in hemodynamic parameters, blood gas analysis, or laboratory analysis as illustrated in Tables 1 and 2. In cerebral microdialysis at baseline, glucose was 0.43 (0.16–0.63) in G_Short_ versus 0.71 (0.54–0.83) mmol/l (*p* =0.03) in G_Long._ Lactate was 1.62 (1.16–1.92) in G_Short_ compared to 2.33 (1.82–3.03) mmol/l (*p* < 0.01) in G_Long_. Moreover, glycerol was 47 (26–50) in G_Short_ compared to 56 (52–63) μmol/l in G_Long_ (*p* < 0.01). No differences were found between groups in levels of pyruvate, glutamate, or lactate/pyruvate ratio (Table 2).

**Table 1:** Hemodynamic parameters for all the animals (*n*=12), divided into Group_Short_ (*n*=6) and Group_Long_ (*n*=6) after cardiopulmonary resuscitation (CPR), followed by extracorporeal pulmonary resuscitation (ECPR)

	ETCO_2_	Baseline	*p* -value	CPR	CPR	CPR	*p* -value	ECPR	ECPR	ECPR	*p* -value
				start	mean	end		30	60	180	
ETCO_2_ (mmHg)	Short	N/A	N/A	11 (11–13)	10 (10–12)	9.0 (8.3–9.0)	0.15	N/A	N/A	N/A	N/A
	Long	N/A		16 (14–17)	15 (12–18)	10 (9–10)		N/A	N/A	N/A	
MAP_Carotis_ (mmHg)	Short	101 (79–106)	0.57	30 (28–31)	32 (32–33)	34 (32–33)	<0.05	81 (64–89)	74 (59–85)	70 (65–94)	0.29
	Long	85 (81–97)		28 (27–33)	31 (29–33)	26 (23–27)		55 (52–62)	50 (48–51)	65 (53–72)	
MAP_Femoralis_ (mmHg)	Short	99 (76–100)	0.69	N/A	N/A	N/A	N/A	59 (46–71)	52 (39–69)	58 (56–71)	0.09
	Long	85 (75–96)		N/A	N/A	N/A		40 (34–47)	38 (32–44)	42 (33–59)	
ICP (mmHg)	Short	12 (10–14)	0.47	17 (15–18)	17 (14–17)	15 (12–16)	0.85	12 (10–15)	13 (11–16)	16 (14–17)	0.43
	Long	12 (11–14)		16 (12–17)	13 (11–17)	10 (10–17)		16 (13–19)	15 (13–19)	15 (13–27)	
CPP (mmHg)	Short	92 (66–93)	0.38	15 (11–15)	18 (15–18)	22 (17–23)	0.10	71 (52–76)	62 (46–73)	57 (50–78)	0.34
	Long	75 (67–85)		15 (11–18)	16 (14–17)	13 (10–13)		42 (34–50)	37 (30–40)	49 (32–59)	
Carotid blood flow (ml/min)	Short	258 (162–309)	0.94	35 (20–36)	19 (14–22)	7 (5.5–9.3)	0.02	103 (91–154)	101 (86–168)	119 (94–207)	0.39
	Long	196 (156–299)		36 (22–39)	27 (18–30)	17 (9.5–22)		94 (75–136)	111 (104–145)	141 (122–198)	
Urine production (ml/h)	Short	N/A	N/A	N/A	N/A	N/A	N/A	210 (133–400)	450 (183–740)	230 (91–750)	<0.01
	Long	N/A		N/A	N/A	N/A		7 (1.0–12)	67 (32–103)	225 (38–315)	
CI/ECMO flow (l/min/m^2^)	Short	3.0 (2.7–3.7)	0.70	N/A	N/A	N/A	N/A	3.1 (2.7–3.6)	3.0 (2.7–3.7)	3.0 (2.7–3.6)	0.97
	Long	3.3 (3.0–3.7)		N/A	N/A	N/A		3.2 (2.8–3.9)	3.2 (3.1–3.7)	3.3 (3.0–3.7)	

Values presented as median, with inter-quartile range in brackets

*ETCO*_*2*_ end-tidal carbon dioxide, *MAP* mean arterial pressure, *CVP* central venous pressure, *ICP* intracranial pressure, *CPP* cerebral perfusion pressure, *CI* cardiac index, *DO*_*2*_*I* oxygen delivery index, *VO*_*2*_*I* oxygen uptake index

**Table 2:** Blood gas analysis, laboratory analysis, and cerebral microdialysis for all the animals (*n*=12), divided into Group_Short_ (*n*=6) and Group_Long_ (*n*=6) after cardiopulmonary resuscitation (CPR), followed by extracorporeal pulmonary resuscitation (ECPR)

	ETCO_2_	Baseline	*p* -value	CPR	CPR	CPR	*p* -value	ECPR	ECPR	ECPR	*p* -value
				start	mean	end		30	60	180	
aB-pH	Short	7.54 (7.51–7.55)	0.94	7.54 (7.52–7.55)	7.54 (7.51–7.55)	7.70 (7.56–7.74)	<0.01	7.33 (7.31–7.34)	7.35 (7.32–7.38)	7.41 (7.35–7.46)	<0.01
	Long	7.54 (7.52–7.56)		7.54 (7.52–7.56)	7.42 (7.31–7.53)	7.17 (7.11–7.40)		7.26 (7.25–7.30)	7.33 (7.30–7.35)	7.43 (7.40–7.47)	
aB-SBE (mmol/l)	Short	5.2 (3.0–6.3)	0.94	5.2 (3.0–6.3)	−2.7 (–3.8 to –1.3)	−3.8 (–6.8 to –1.3)	<0.01	−5.1 (–6.3 to –4.9)	−4.4 (–5.2 to –3.7)	1.3 (–1.6–2.2)	<0.01
	Long	4.9 (3.9–6.0)		4.9 (3.9–6.0)	−13 (–14 to –10)	−16 (–18 to –13)		−8.7 (–8.9 to –6.3)	−6.0 (–7.1 to – 4.8)	1.5 (1.0–5.7)	
aB-Lactate (mmol/l)	Short	2.8 (2.4–4.8)	0.81	2.8 (2.4–4.8)	6.9 (4.2–7.9)	7.9 (4.3–9.1)	<0.01	10 (8.7–11)	9.6 (9.011)	5.1 (4.3–6.6)	<0.01
	Long	3.2 (2.8–3.8)		3.2 (2.8–3.8)	12 (11–14)	14 (14–16)		15 (14–16)	15 (14–16)	7.3 (5.4–8.3)	
P-NGAL (ug/l)	Short	92 (86–94)	0.75	N/A	N/A	95 (72–112)	0.13	107 (87–145)	114 (84–152)	138 (97–162)	0.04
	Long	89 (86–99)		N/A	N/A	128 (109–141)		202 (181–220)	220 (170–274)	324 (206–411)	
P-S100B (ng/l)	Short	0.40 (0.32–0.48)	0.80	N/A	N/A	0.60 (0.60–1.05)	0.09	0.70 (0.62–0.78)	0.55 (0.42–0.60)	0.30 (0.23–0.45)	0.12
	Long	0.40 (0.32–0.55)		N/A	N/A	1.15 (0.92–1.45)		1.15 (1.10–1.20)	0.75 (0.70–0.80)	0.60 (0.52–0.60)	
Lactate (mmol/l)	Short	1.6 (1.2–1.9)	<0.01	1.6 (1.2–1.9)	2.2 (1.6–2.9)	2.2 (1.6–2.9)	<0.01	3.3 (3.0–3.7)	2.1 (1.4–2.5)	0.85 (0.7–1.1)	0.25
	Long	2.3 (1.8–3.0)		2.3 (1.2–3.0)	4.9 (4.2–5.5)	5.1 (4.9–5.3)		3.35 (2.4–4.2)	2.3 (1.7–2.8)	0.70 (0.6–1.1)	
Pyruvate (μmol/l)	Short	0.04 (0.02–0.06)	0.45	0.04 (0.02–0.06)	0.04 (0.02–0.04)	0.04 (0.02–0.04)	0.90	0.10 (0.09–0.12)	0.08 (0.05–0.08)	0.03 (0.03–0.04)	0.68
	Long	0.05 (0.04–0.05)		0.05 (0.04–0.05)	0.03 (0.03–0.04)	0.03 (0.03–0.04)		0.06 (0.06–0.07)	0.07 (0.05–0.07)	0.03 (0.03–0.04)	
Lactate/pyruvate ratio	Short	38 (29–74)	0.08	38 (29–74)	60 (44–67)	60 (44–67)	<0.01	31 (30–38)	29 (25–31)	28 (21–35)	0.43
	Long	44 (40–69)		44 (40–69)	129 (117–147)	152 (129–186)		46 (40–56)	39 (33–42)	22 (15–42)	
Glycerol (μmol/l)	Short	47 (26–50)	<0.01	47 (26–50)	37 (31–42)	37 (31–42)	<0.01	61 (58–68)	66 (50–71)	23 (19–26)	<0.01
	Long	56 (52–63)		56 (52–63)	58 (55–62)	65 (59–70)		90 (87–99)	93 (77–111)	36 (31–42)	
Glutamate (μmol/l)	Short	24 (17–40)	0.12	24 (17–40)	29 (16–33)	29 (16–33)	0.02	7.2 (4.9–12)	7.2 (5.1–9.4)	5.4 (3.3–6.2)	0.82
	Long	55 (42–72)		55 (42–72)	67 (59–70)	71 (53–89)		8.2 (6.9–14)	6.4 (5.4–8.2)	4.3 (4.2–6.5)	

*ETCO*_*2*_ end-tidal carbon dioxide, *aB* arterial blood, *SBE* standard base excess, *AVO*_*2*_ arterio-venous oxygen difference, *P-NGAL* plasma neutrophil gelatinase–associated lipocalin, *P-S100B* plasma calcium binding protein B

Values presented as median, with inter-quartile range in brackets

### CPR

#### Hemodynamic Parameters

The mean time of CPR was 11.6 (9–15) min in G_Short_ (*n*=6) and 35.5 (27–45) min in G_Long_ (*n*=6).

**Fig. 1:**
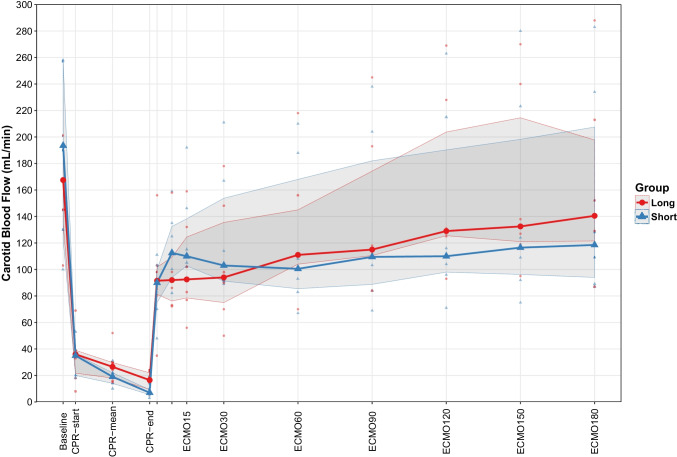
Carotid blood flow during the experiment. At the end of ECPR 180 min, *p* =0.29. Grey-shaded area represents inter-quartile range

Carotid blood flow remained higher in G_Long_ during CPR and was 17 (9.5–22) ml/min compared with 7 (5.5–9.3) ml/min in G_Short_ at end of CPR (*p* =0.02) (Table 1 and Fig. 1). Mean arterial blood pressure was lower in G_Long_ at 26 (23–27) mm Hg versus 34 (32–33) mmHg in G_Short_ (*p* < 0.05) (Table 1). Cerebral perfusion pressure was comparable between G_Long_ and G_Short_ throughout CPR (Table 1).

#### Arterial Blood (B)

**Fig. 2:**
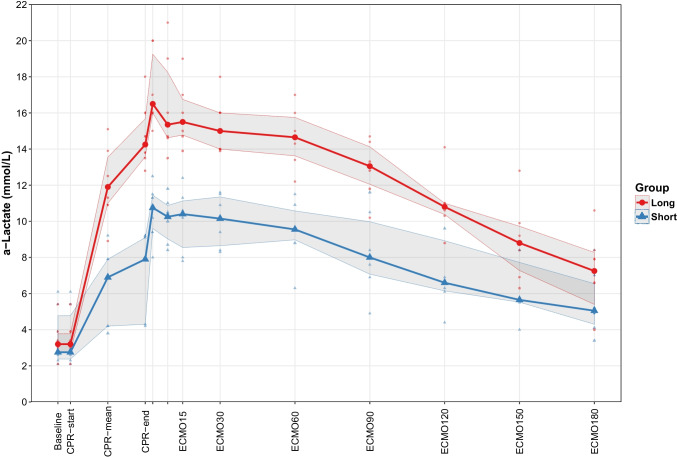
Levels of B-lactate during the experiment. At the end of ECPR 180 min, *p* < 0.01. Grey-shaded area represents inter-quartile range

At end of CPR, B-lactate was lower in G_Short_ at 7.9 (4.3–9.1) compared to 14 (14–16) in G_Long_ (*p* < 0.01) (Table 2 and Fig. 2).

#### Microdialysis

**Fig. 3:**
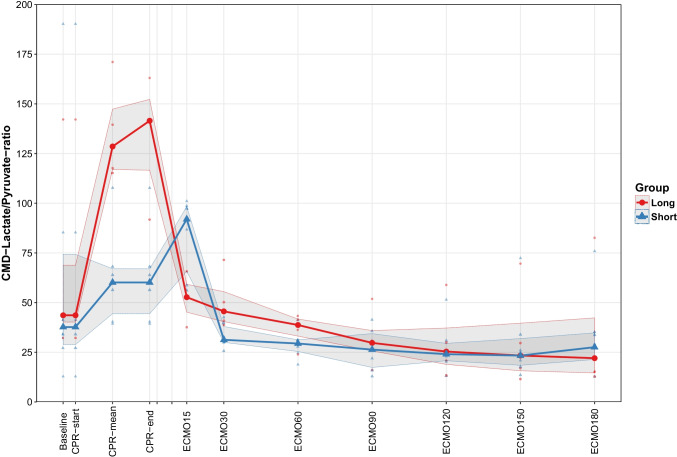
Lactate/pyruvate ratio during the experiment. At the end of ECPR 180 min, *p* =0.43. Grey-shaded area represents inter-quartile range

At end of CPR, lactate was higher in G_Long_ at 5.1 (4.9–5.3) compared to G_Short_ at 2.2 (1.6–2.9) mmol/l (*p* < 0.01) (Table 2). Moreover, the lactate-pyruvate ratio was higher in G_Long_ at 152 (129–186) compared with G_Short_ at 60 (44–67) (*p* < 0.01) (Fig. 3). In addition, glycerol was higher in G_Long_ at 65 (59–70) versus 37 (31–42) μmol/l in G_Short_ (*p* < 0.01) and glutamate at 71 (53–89) in G_Long_ versus 29 (16–33) μmol/l in G_Short_ (*p* =0.02) (Table 2).

### End of ECPR

#### Arterial Blood (B)

B-Lactate fell in both groups and was 5.1 (4.3–6.6) in G_Short_ versus 7.3 (5.4–8.3) mmol/l in G_Long_ (*p* <0.01), as depicted in Table 2 and Fig. 2.

#### Hemodynamic Parameters

Carotid blood flow was restored during ECPR in G_Short_ and there were no differences in cerebral perfusion pressure or any of the hemodynamic parameters between the two groups during and at the end of ECPR (Table 1, Figs. 1 and 2).

#### Kidney

Throughout ECPR, urinary output was significantly higher in G_Short_ (*p* < 0.01); however, the rate of flow approached each other and was comparable. Moreover, P-nGAL was higher in G_Long_ at 324 (206–411) μg/l compared with 138 (97–162) μg/l in G_Long_ (*p* =0.04) (Table 2). No differences were found in renal histopathology post-ECPR between the two groups.

#### Brain

**Fig. 4:**
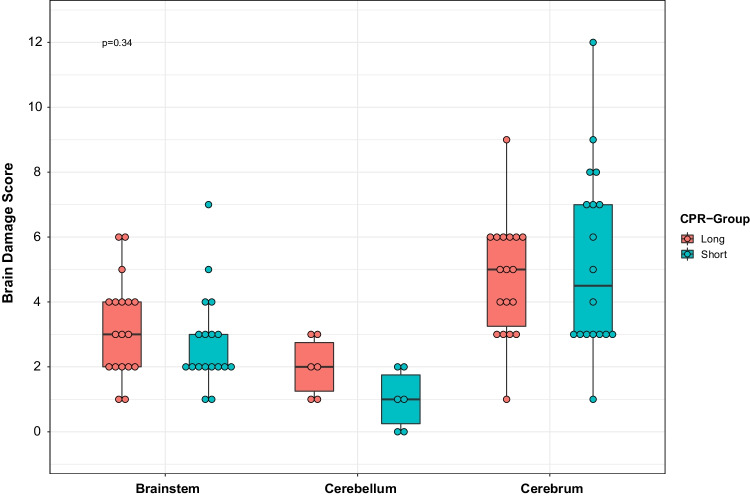
Box plot of brain damage score, comparison of the two groups, *p* =0.34

There was no difference in levels of P-S100B or in the brain damage score between the two groups at the end of ECPR (Table 2 and Fig. 4). The difference in lactate/pyruvate ratio returned to normal after the episode of ECPR (Fig. 3), and no other differences were detected in the parameters of microdialysis at end of ECPR (Table 2).

## Discussion

This experimental model of conventional CPR followed by ECPR used ETCO_2_ as the determinant of when to start ECPR. The main finding is that when levels of ETCO_2_ are used to initiate ECPR, there were some subtle biochemical differences, but the extent of kidney and brain injury in histopathology was comparable between the groups at end of ECPR, regardless of the time of CPR.

The chances of survival are low when ETCO_2_ is less than 10 mmHg measured at 20 min of conventional CPR [13–15]. The French ministry of health recommends against ECPR if ETCO_2_ is below 10 mmHg [16]. Moreover, ECPR is also associated with worse results if CPR is performed for a long time. Obviously, CPR time should not be disregarded when deciding upon the initiation of ECPR treatment, as illustrated by several retrospective studies on predictive factors [8, 9, 17]. On the other side of the spectrum, time is of less importance if CPR is poorly performed. This implicates that time alone should not be used to include or exclude patients from ECPR treatment. Optimal CPR only corresponds to approximately 30% of pre-arrest cardiac output, and there have been many reports of successful ECPR treatments that significantly exceed recommended time limits for CPR [18, 19]. In summary, there is a need to identify clinically relevant factors as a complement to time that reflects the current physiological condition of the resuscitated patient. It is possible that ETCO_2_ can be such a factor.

### Blood Gas Parameters

B-Lactate was higher in G_Long_ compared with G_Short_ at end of CPR and ECPR. The clinical relevance of these parameters is not possible to define in our study. In a recent study by Jung et al. [20], clearance of lactate in ECPR patients was found to be predictive of neurological outcome, but not survival. Its role in decision-making on ECPR treatment has yet to be more precisely determined.

### Brain

ETCO_2_ during CPR is known to correlate with cardiac output and cerebral macro- and microvasculature flow [7, 14, 21]. To describe early metabolic changes and to increase the temporal resolution of our findings, we chose to complement other findings with the use of cerebral microdialysis. In this study, cerebral microdialysis suggests suboptimal circulation with impaired metabolic conditions in both groups during CPR, although significantly worse in the group with longer CPR. This is evident by a considerable rise in the lactate-pyruvate ratio and level of glutamate at induction of cardiac arrest and is in accordance with other studies on microdialysis and cardiac arrest [22–24].

All parameters of microdialysis returned to baseline or sub-baseline values during ECPR. At the end of the experiment, differences between groups were eradicated, except for that regarding glycerol. This could be interpreted as if cerebral metabolism is, at least partly, restored by extracorporeal treatment. However, microdialysis is just a sample from a single locus of the brain, which is why having a means to measure global injury is imperative to strengthen our results.

S100B is a glia-specific protein, and an established peripheral biomarker of CNS injury, and has been shown to predict neurologic outcomes after resuscitation of cardiac arrest and ECMO patients [25–27]. In a porcine CPR model by Zhang et al. [28], a significant increase of S100B was recorded after cardiac arrest, matching the degree of histopathological findings. In our study, levels of S100B were overall lower and returned to baseline at end of ECPR. A somewhat optimistic interpretation could be that the metabolic changes seen during CPR become of less clinical importance if they can be reversed by ECPR in a reasonably short time frame. Moreover, one might argue that an alternative method to detect early signs of ischemia should have been used. Suchalodskiene et al. [29] suggest the analysis of mitochondrial oxidative phosphorylation, where a decrease occurs before histological signs of neuronal death can be detected. Although not valid for all areas of the brain, microdialysis can be considered as its substitute in the present study.

### Kidney

The biomarker NGAL, proven to rise within 3 h of AKI [30], predicts AKI in the adult cardiac surgery patient, critically ill, and kidney transplant patient [31–33]. NGAL rose and kept rising in G_Long,_ consistent with lower urinary output. Our findings of NGAL and urinary output levels are in accordance with those found by others [34] who also showed histological derangement after ROSC in a similar porcine model. Clearly, the group with longer CPR had more pronounced renal impairment, measured as raised NGAL and decreased urinary output. Diuresis was eventually restored upon extracorporeal circulation. However, no differences were observed in pathology damage score between the groups and probably the time hypoperfusion should have been longer for more sustainable histological changes. This supports the notion that the level of ETCO_2_ as a complement to time is relevant for the initiation of ECPR. In this study, the time interval was between 11 and 45 min before ECPR was initiated. Following cardiac arrest, 11 min is considered a short time. However, at this time, some of the pigs will already be severely deranged, while others can last until 45 min before deterioration starts.

The clinical significance of kidney histopathology findings is unclear, but it is known that the renal medulla is sensitive to hypoxia and hypotension, and that renal impairment in the ECPR setting is a negative prognostic factor [35, 36]. However, longer times of hypoperfusion have shown to be needed for histological findings to appear [37]. Also, in our previous study, a correlation between ETCO_2_ during CPR and kidney injury following ECPR [38] strengthens the role of ETCO_2_ as a guide.

### Clinical Relevance

The cornerstone of a favourable outcome after ECPR remains patient selection, preferably based on prognostic factors. Unfortunately, evidence is lacking to give firm guidance on the CPR to ECPR transit. Although this study is of an experimental nature, we believe that the finding of ETCO_2_ relevance can add some information regarding the selection of ECPR candidates and the timing of ECPR in addition to CPR time.

### Study Limitations

First, the small sample size in each group must be considered. The statistical methods are chosen to compensate for the small numbers. Secondly, the limited time of ECPR must be considered, as it can be argued that 3 h is too short to detect significant histological changes in the brain and kidney with our chosen methods. Also, differences in glucose, lactate, and glycerol between groups were seen in baseline. This might partly be explained by allowing limited time (less than 1 h) to stabilize values before starting measurement. The physiological status of each animal when arriving at the laboratory is one factor hard to influence; the animals have been treated in accordance with good animal care; however, nutritional status and fluid balance are unknown and could theoretically affect baseline parameters.

Furthermore, the preparation of microdialysis is known to affect lactate and glucose to a certain extent and might influence differences. Moreover, in the baseline statistics, mixed effect models were not used and could also explain differences between the groups. However, in the longitudinal measurements during CPR and ECMO, mixed effect models were used, and these methods take inter-individual differences into account. Finally, the clinical significance of the findings cannot be determined as subjects were not awakened.

## Conclusion

Cerebral microdialysis indicates a time-dependent risk of ischemic injury, which is restored during ECMO. No apparent histological differences of tissue damage in brains or levels of S100B in plasma were detected between groups. This might suggest that ETCO_2_ could be used as a marker for brain injury following ECPR and supports its use as a complement to time of CPR as an eligibility criterion for ECPR.
